# Insight on Genes Affecting Tuber Development in Potato upon *Potato spindle tuber viroid* (PSTVd) Infection

**DOI:** 10.1371/journal.pone.0150711

**Published:** 2016-03-03

**Authors:** Konstantina Katsarou, Yun Wu, Runxuan Zhang, Nicola Bonar, Jenny Morris, Pete E. Hedley, Glenn J. Bryan, Kriton Kalantidis, Csaba Hornyik

**Affiliations:** 1 Institute of Molecular Biology and Biotechnology, Foundation for Research and Technology, Heraklion, Crete, Greece; 2 Department of Horticulture, College of Agriculture and Biotechnology, Zhejiang University, Hangzhou, China; 3 Information and Computational Sciences, The James Hutton Institute, Invergowrie, Dundee, United Kingdom; 4 Department of Biology, University of Crete, Heraklion, Crete, Greece; 5 Cell and Molecular Sciences, The James Hutton Institute, Invergowrie, Dundee, United Kingdom; Agriculture and Agri-Food Canada, CANADA

## Abstract

Potato (*Solanum tuberosum L*) is a natural host of *Potato spindle tuber viroid* (PSTVd) which can cause characteristic symptoms on developing plants including stunting phenotype and distortion of leaves and tubers. PSTVd is the type species of the family *Pospiviroidae*, and can replicate in the nucleus and move systemically throughout the plant. It is not well understood how the viroid can affect host genes for successful invasion and which genes show altered expression levels upon infection. Our primary focus in this study is the identification of genes which can affect tuber formation since viroid infection can strongly influence tuber development and especially tuber shape. In this study, we used a large-scale method to identify differentially expressed genes in potato. We have identified defence, stress and sugar metabolism related genes having altered expression levels upon infection. Additionally, hormone pathway related genes showed significant up- or down-regulation. *DWARF1/DIMINUTO*, *Gibberellin 7-oxidase* and *BEL5* transcripts were identified and validated showing differential expression in viroid infected tissues. Our study suggests that gibberellin and brassinosteroid pathways have a possible role in tuber development upon PSTVd infection.

## Introduction

Viroids are non-coding RNA pathogens with unencapsidated, circular RNA genomes (reviewed by [1]) which are relatively short (246–467 nt), and which cause plant diseases of important crops including tomato and potato [2]. Viroids were reported and described first in 1970’s in the spindle tuber disease of potato [3]. Viroids spread mechanically which is facilitated by harvesting or cultural operations. Additionally, their transmission by seed and pollen have been proposed [4]. *Potato spindle tuber viroid* (PSTVd) is the type member of the *Pospiviroidae* family of viroids [5]. PSTVd contains five distinct domains (central, pathogenic, variable and two terminal domains). Its replication occurs in the nucleus via an asymmetric rolling cycle mechanism. The circular viroid RNA (+) is transcribed into (-) RNA which acts as a template of DNA-dependent RNA polymerase II (RNAP II) complex for the synthesis of (+) RNA [6–8].

Symptom development is strongly influenced by the viroid genomic RNA structure/sequence, the host and the environment. Symptoms vary from mild to severe and can affect either the entire plant or specific organs only, such as leaves, fruits, flowers, roots and storage organs [2]. PSTVd can infect ornamental plants (*Solanaceae* or *Asteraceae*) and can also cause severe symptoms in crops such as tomato and potato [9]. Typical PSTVd symptoms are stunting, shortening of internodes and petioles, severe epinasty and wrinkling of leaves. Tubers show elongation upon infection, with the appearance of prominent bud scales/eyebrows and sometimes growth cracks [10]. Since viroids do not encode proteins, their replication and infection depend on host proteins [1]. However, viroid infection also has a global effect on plant gene expression. Tomato plants were extensively studied for genes which are directly or indirectly affected upon viroid infection [11–13]. Differentially expressed genes were identified for defence /stress response, cell wall structure, chloroplast function, protein metabolism and hormone signalling pathways, all of which can potentially affect different aspects of plant development.

Tuberization is a specific developmental process when an underground modified stem (stolon) starts to differentiate and swell, to develop the tuber which is unique to some *Solanum* species [14]. Potato originates from South America where it is adapted to short day and relatively low night temperatures which can induce tuber formation [15]. In the photoperiod induced tuberization pathway day length can induce tuber formation starting with the perception of signal in leaves via *phytochrome B* (*phyB*), leading to expression of *CYCLING DOF FACTOR 1* (*CDF1*) which can influence the downstream genes *CONSTANS* (*CO*) and *FLOWERING LOCUS T* (*FT*) family members (*StSP5G* and *StSP6A*), and *StSP6A* can induce tuber differentiation in stolons as the mobile signal [16]. Additionally, non-coding RNAs (micro RNAs) were implicated as having a role in tuber formation including miR156 and miR172 [16, 17]. Modern potato cultivars were selected against the photoperiodic control for the ability to tuberize under long day conditions [14]. BEL5 protein, with its partner POTH1, was reported to induce tuber formation by mediating hormone levels in stolon tips [18]. Recent studies showed how *BEL5* trancript can move in the phloem with the help of polypyrimidine tract-binding proteins (StPTB1 and StPTB6) and the importance of BEL5 protein in tuber formation via the regulation of *StSP6A* [19, 20]. Hormones have fundamental roles in tuberization [21] and it was reported that gibberellins (GAs) have negative impact on tuber formation [22, 23], and auxin and strigolactones (SLs) can alter tuber development [24, 25]. Recently, cytokinins (CKs) were proposed to induce aerial minitubers in tomato, similar to potato tubers [26].

Tuber shape was investigated previously but it is still not known which genes are responsible for the tuber shape trait. Initially, it was explained by a single locus *Ro* on chromosome 10, with round being dominant over long [27]. Other genetics studies identified several Quantitative Trait Loci (QTL) on different chromosomes affecting tuber shape [28–31]. Our previous study identified two QTL on chromosome 2 and 10 for tuber shape; the chromosome 10 QTL can explain the higher variation in the population as seen in other studies. Additionally, we found that tuber shape is determined in the early stages of tuber development [32]. PSTVd infection causes a severe tuber phenotype, which leads to the change of shape, and a characteristic elongation of the tuber [10]. Thus, we hypothesized that infection could lead to the regulation of one of the aforementioned genes, which will alter tuber shape or could help us identify new unknown candidates.

In this study we have used a custom 60k microarray to identify differentially expressed genes in PSTVd-infected potato. This is one of the first studies using a high-throughput approach to analyse genome-wide gene expression in viroid-infected potato. The potato cultivar Safari (*Solanum tuberosum L* ‘Safari’) was infected with a severe strain of PSTVd, and leaf and tuber tissues were analysed at early and late time points of infection. Genes connected to stress responses, defence pathways, sugar metabolism and hormone signalling pathways showed differential expression. The microarray data was validated through independent expression analysis of selected genes and we found evidence that the gibberellin and brassinosteroid pathways might have a key role in tuber/tuber shape formation upon infection.

## Results

### PSTVd infection of potato plants

**Fig 1:**
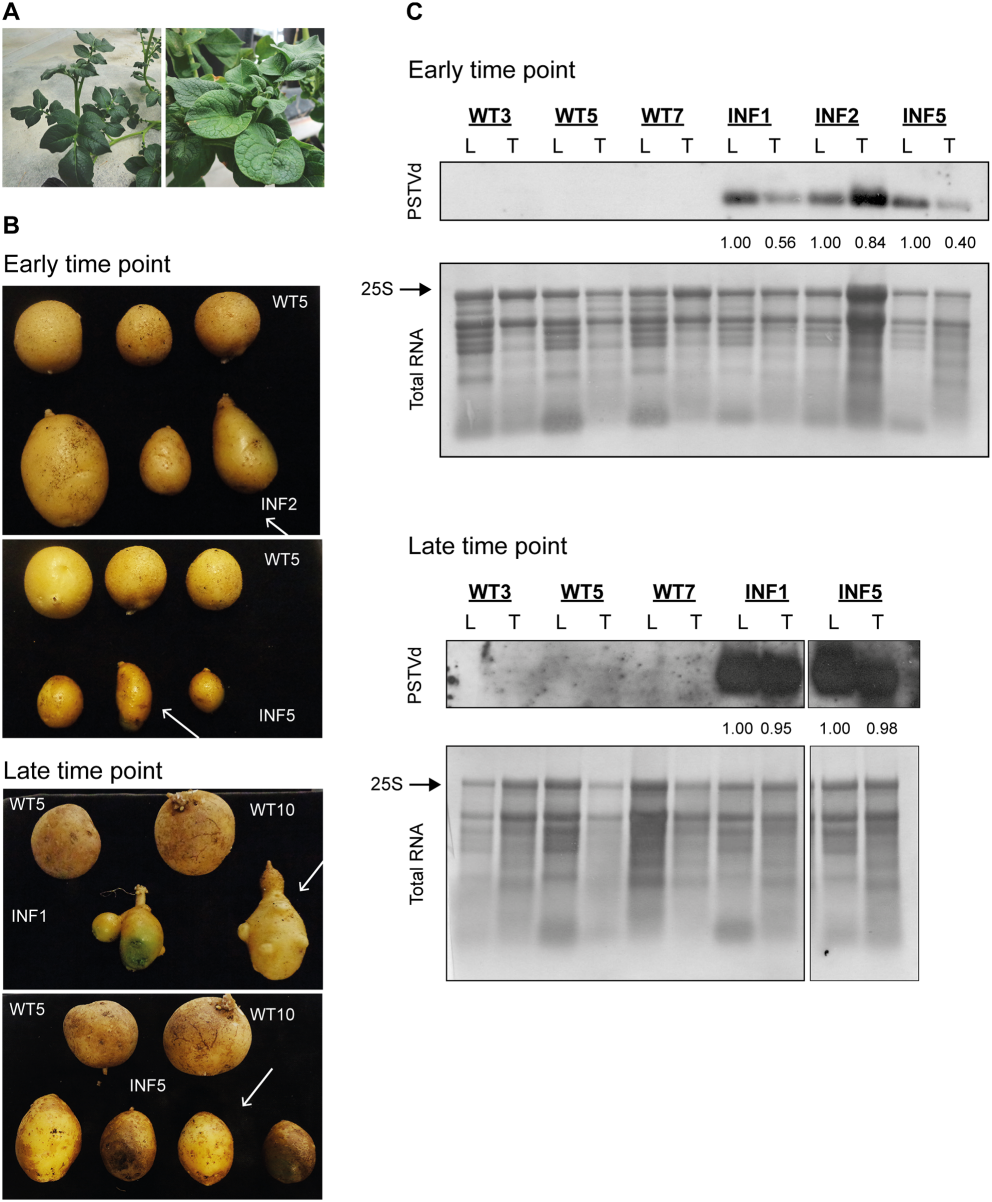
Symptom development and PSTVd accumulation in infected potato plants. (A) Mock inoculated Safari plant (right) and PSTVd infected plant (left) at 14 weeks post-inoculation (wpi). (B) Tubers of mock inoculated (WT) and infected (INF) plants at 14 wpi on the upper panels and 21 wpi on the lower panels. Numbers indicate the different individual plants. White arrows show which tubers were used for RNA extraction. (C) Northern blot analysis of mock inoculated (WT) and infected (INF) plants. Numbers indicate different individual plants. Upper panel shows viroid accumulation in early (14 wpi) samples and lower panel shows the presence of PSTVd in late (21 wpi) samples. Methylene blue staining of ribosomal RNAs was used as loading control; the arrow shows the 25S ribosomal RNA which was used for normalization. The quantification of viroid signal is shown as numbers under the viroid RNA gel blot panels. Relative normalisation was performed for each plant; normalised to leaf viroid level using ribosomal RNA as control.

To examine the effect of PSTVd infection on tuber development we selected the potato cultivar Safari, which produces round tubers. Potato tubers were planted into pots in a glasshouse and 3.5 week old plants (4–5 leaf stage) were infected through mechanical inoculation with *in vitro* transcripts of PSTVd^KF440-2^ [33]. Infection was confirmed by RNA gel blot analysis of leaf tissues nine weeks post inoculation (wpi). As shown in S1 Fig, the majority of plants were infected by PSTVd. In addition, infected plants showed typical visible symptoms of viroid infection: decrease in shoot and internode length, shortening of petioles, and distortion of leaves including wrinkling (Fig 1A). Two different time points were chosen, referred to as ‘early’ (14 wpi) and ‘late’ (21 wpi), in order to address the effect of the infection on tuber development. In both developing (early) and fully developed (late) tuber stages infected plants showed a change in shape from rounded to elongated without any obvious size changes compared to the non-infected plants. This effect was further pronounced at the late time point when deeper eyes and occasionally lumps on the tubers were observed (Fig 1B). Upper leaves of three infected plants as well as tubers of the same plants were collected at early and late points and analysed for the presence of the viroid. Fig 1C shows that infected plants had high levels of viroid RNA while the control plants were viroid free. Infection seems to increase through time, since more PSTVd accumulated in both tissues at late sampling. However, tubers seem to accumulate less PSTVd compared to leaves, especially at early sampling. It should be noted that due to technical problems at the late time point only two plants were further analysed (Fig 1C).

### Transcriptome changes upon PSTVd infection

**Fig 2:**
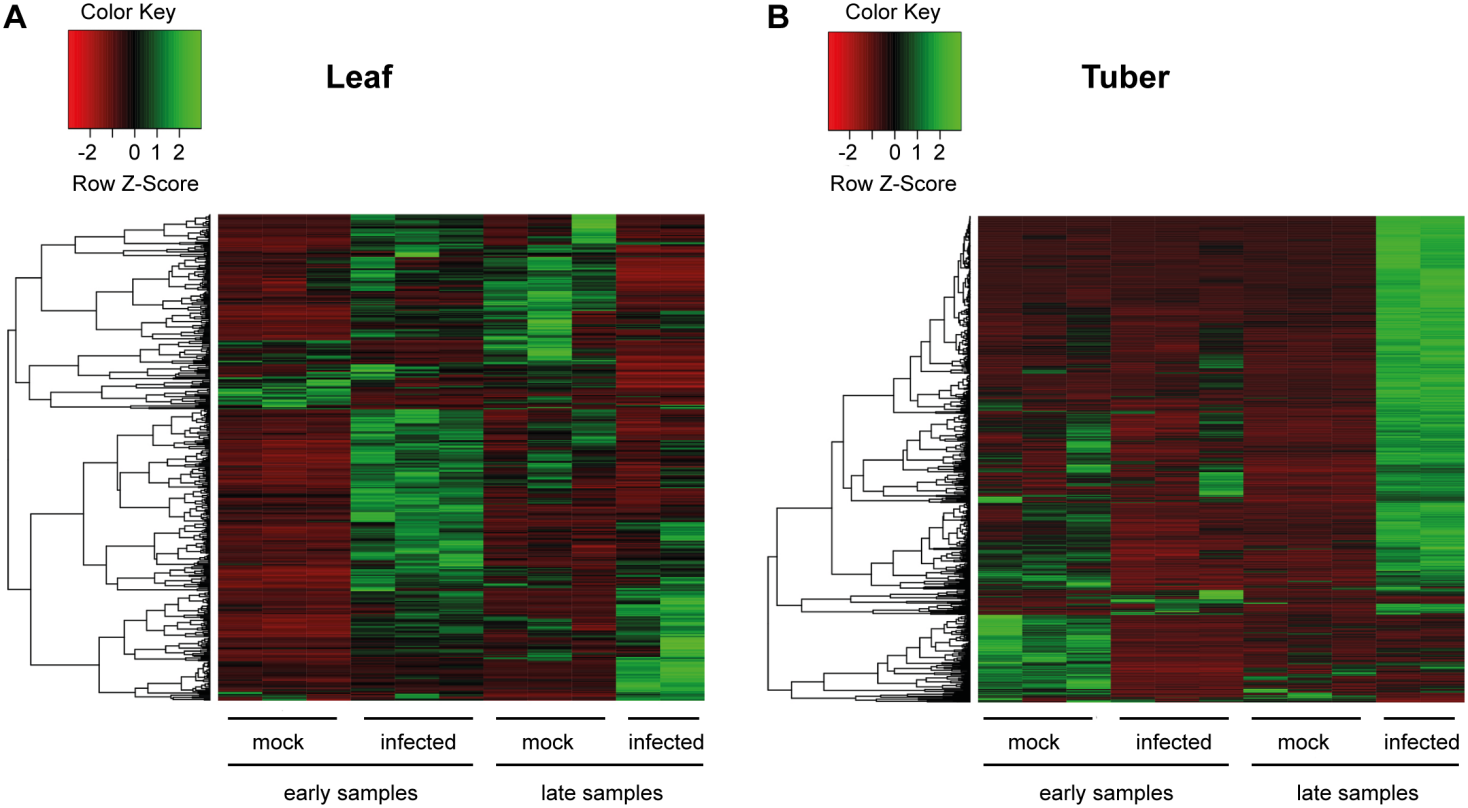
Heatmap representation of differentially expressed genes upon PSTVd infection. Visualization of differentially expressed genes of leaf (A) and tuber (B) tissues in mock inoculated and infected plants as indicated. Three biological replicates are shown, but for late infected tissues only two. Colour key indicates the expression change compared to mock inoculated samples.

In order to identify differentially expressed genes during the early and late stages of PSTVd infection in potato a custom 60k potato microarray (Agilent Technologies) was used. This platform enabled us to study the gene expression levels at high throughput with good confidence. Total RNA extracted from leaf and tuber tissues at early and late stages were analysed. Genes which show increased (≥2 fold) or decreased (≤0.5 fold) expression levels were identified (p-value ≤0.05) and can be found in S1 Table. As expected, upon viroid infection many genes (1226 genes) showed altered expression. In early leaf samples the majority of genes (268 genes) which show differential expression are upregulated in infected tissues (Fig 2A). These genes encode mainly heat-shock proteins and transcription factors, along with genes connected to hormone regulation and those involved in defence pathways (NBS-LRR resistance proteins, peroxidases). In contrast, at the later stage of infection fewer genes (63 genes) show up-regulation in infected leaves and a larger number (94 genes) are down-regulated (Fig 2A). The affected genes at this stage are those with stress response, genes involved in sugar metabolism and some transcription factors. In contrast to the early leaf samples, early tuber tissues show down-regulation for many genes (185 genes; Fig 2B). There is a clear clustering of biological replicates from mock-inoculated and infected tissues. Genes encoding disease resistance proteins (like Cathepsin B-like cysteine proteinase), oxidases and those connected to hormone regulation show strong change in expression upon infection (S1 Table). Auxin and gibberellin related genes (Auxin growth promotor protein, Gibberellin 7-oxidase) are down-regulated in infected tissues suggesting that the tuber development control via hormones might be compromised upon infection. We found the strongest changes between mock inoculated and infected tissues in the case of late tuber samples. A heat map shows that the majority of genes showing differential expression are up-regulated in viroid infected tubers (Fig 2B). Many transcription factors (*MYB*, *WRKY*, F-box domain containing protein, transcription factor Jumonji), defence related genes (*TMV* induced protein 1–2, NBS-LRR resistance protein) and different types of kinases show altered gene expression during PSTVd infection. In addition, the *BEL5* transcripts showed up-regulation in late tuber tissues upon infection indicating that tuber development is strongly influenced by the viroid. The expression levels of genes could be interesting in both tissues at different time points. In order to see the expression levels of all the transcripts in potato, we included all the microarray data into S2 Table with normalised values and standard errors.

### Gene ontology enrichment analysis of differentially expressed genes

**Fig 3:**
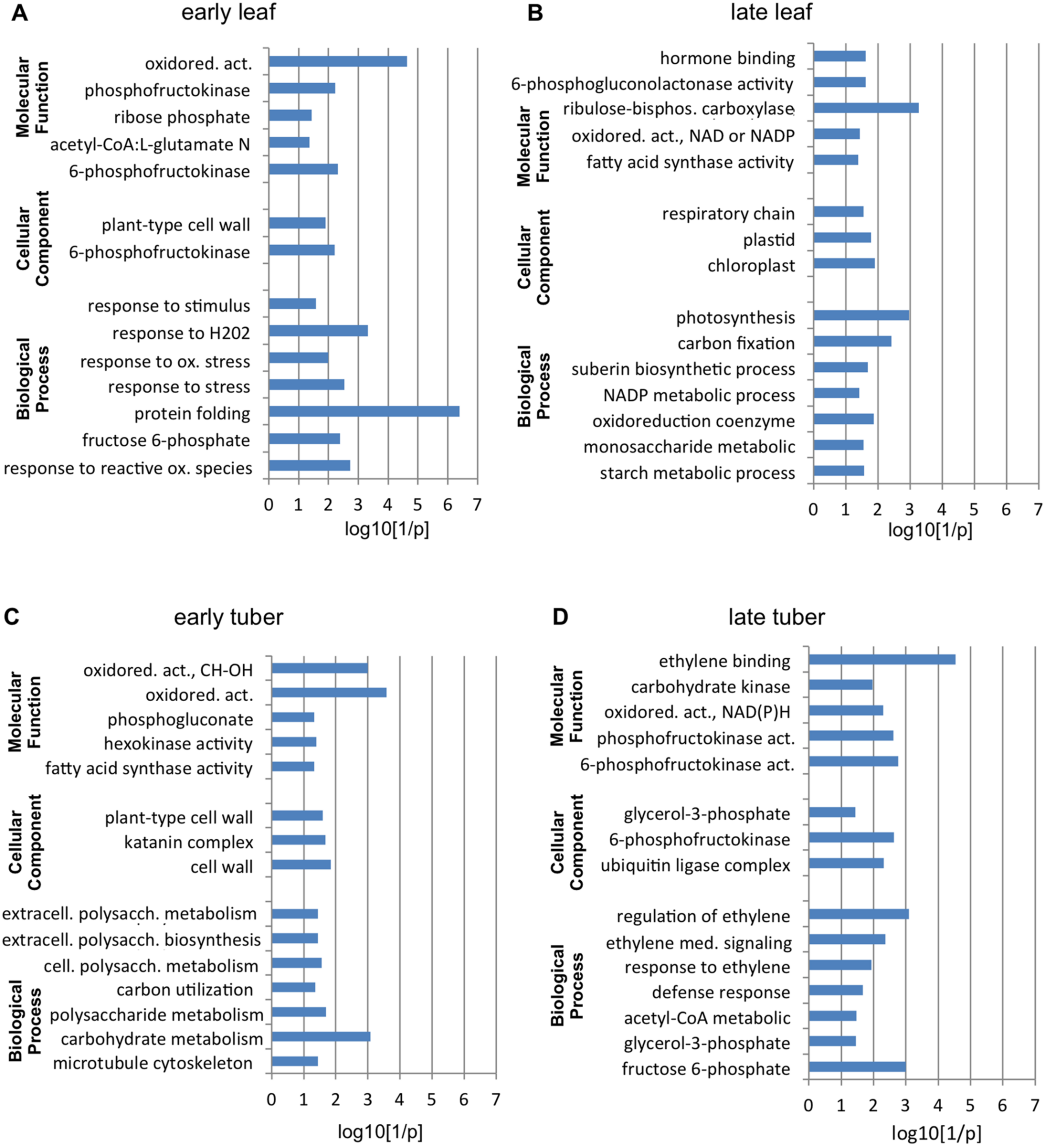
Representation of GO term enrichment. Selected GO term enrichments are visualized in early (A) and late (B) leaf samples. Figs (C) and (D) show GO term enrichment for early and late tuber samples, respectively. Categories are indicated on Y axis, log10[1/p] is on the X-axis where p is the p-value of the enrichment.

To help understand the role of differentially expressed genes among the early/late leaf and tuber tissues we carried out GO term analysis. Analysis was performed to identify GO terms which are significantly enriched in the dataset. Three major GO categories were used in our analysis: ‘Biological Process’, ‘Cellular Component’ and ‘Molecular Function’. All the data can be found in S3 Table. In order to identify significant enrichment we used the p-values (log10[1/p]) of enriched categories and selected some sub-categories for visualization (Fig 3). In early leaf samples there is a significant enrichment for categories in viroid infected tissues which are connected with response to stimulus, oxidative stress and reactive oxygen species. Additionally, the ‘Molecular Function’ category suggests there is a change in gene expression levels of genes related to sugar metabolism (Fig 3A). In late leaf samples genes connected with basic cell metabolism are affected and show significant enrichment: photosynthesis, carbon fixation, hormone binding and sugar/fatty acid metabolism (Fig 3B). Tuber samples show slightly different enrichment for GO terms. Comparing the viroid infected tissues to mock inoculated samples we can see significant enrichment for polysaccharide and carbohydrate metabolic process at the early stage. Additionally, plant-type cell wall and sugar metabolism categories are enriched as well, although the cell wall category shows similar enrichment in early leaf samples (Fig 3C). Analysing late tuber samples there is a significant enrichment for ethylene metabolism (ethylene binding, response to ethylene stimulus, ethylene mediated signalling pathway), sugar metabolism and defence response (Fig 3D). All this data suggest that there are significant expression changes of genes affecting sugar metabolism, starch development and hormone homeostasis of leaf and tuber tissues.

### Analysis of genes affecting tuberization and tuber shape development

The genome-wide expression study using the 60k potato microarray allowed us to examine the expression levels of genes which show differential expression at early and late stages of viroid infection. This is a valuable dataset for finding pathogenesis related genes connected to viroid infection, but our primary focus is on better understanding the altered tuberization, tuber shape development and finding genes affecting these biological processes. Our previous study using a highly heterozygous full-sib diploid potato population (06H1) identified two major loci segregating for tuber shape [32]. We identified two markers on chromosome 2 and 10 under the quantitative trait loci (QTL) which show the highest Kruskal–Wallis (KW) value, indicating the highest influence of these loci on the variability in the population on the length/width (LW) ratio of tubers (shape). These Single Nucleotide Polymorphism (SNP) markers originating from the Infinium 8303 Potato Array are c1_8020 for chromosome 10 and c2_46885 on chromosome 2. We identified the superscaffolds which contain these markers and also the neighbouring superscaffolds. The genes located on these superscaffolds were extracted and examined according to their annotated functions whether they might have a role in tuber shape formation or tuberization (S4 Table). The differentially expressed genes from the viroid microarray data were compared with these sets of genes and candidate genes were identified (Table 1). Interestingly, only two genes show differential expression on the superscaffolds under the QTL on chromosome 10. On the three identified superscaffolds of chromosome 2 there are 8 genes which show differential expression upon viroid infection. Because of the low number of candidate genes and their annotated functions on chromosome 10 we focused further on the genes located on chromosome 2.

**Table 1:** Differentially expressed genes on three superscaffolds under the tuber shape QTLs.

Superscaffold (DMB)	Gene number (DMG)	Start	End	Gene description
Chromosome 2				
PGSC0003DMB000000406	PGSC0003DMG400010362	364889	373913	Cathepsin B-like cysteine proteinase
PGSC0003DMB000000141	PGSC0003DMG400021216	435273	436517	Nectarin 5
PGSC0003DMB000000141	PGSC0003DMG400021196	989253	995484	Charged multivesicular body protein 2b-B
PGSC0003DMB000000141	PGSC0003DMG400021132	1002442	1005225	Cation/calcium exchanger 4
PGSC0003DMB000000141	PGSC0003DMG400021142	1204198	1207368	DWARF1/DIMINUTO
PGSC0003DMB000000141	PGSC0003DMG400021213	1418027	1424568	Argonaute protein group
PGSC0003DMB000000552	PGSC0003DMG400008284	24433	29363	60S ribosomal protein L18a, plant
PGSC0003DMB000000552	PGSC0003DMG400042498	108548	109345	Chlorophyll a/b binding protein
Chromosome 10				
PGSC0003DMB000000385	PGSC0003DMG400031222	410541	417055	Phospholipid-transporting atpase
PGSC0003DMB000000385	PGSC0003DMG400031223	449401	456518	Lung seven transmembrane receptor family protein

**Fig 4:**
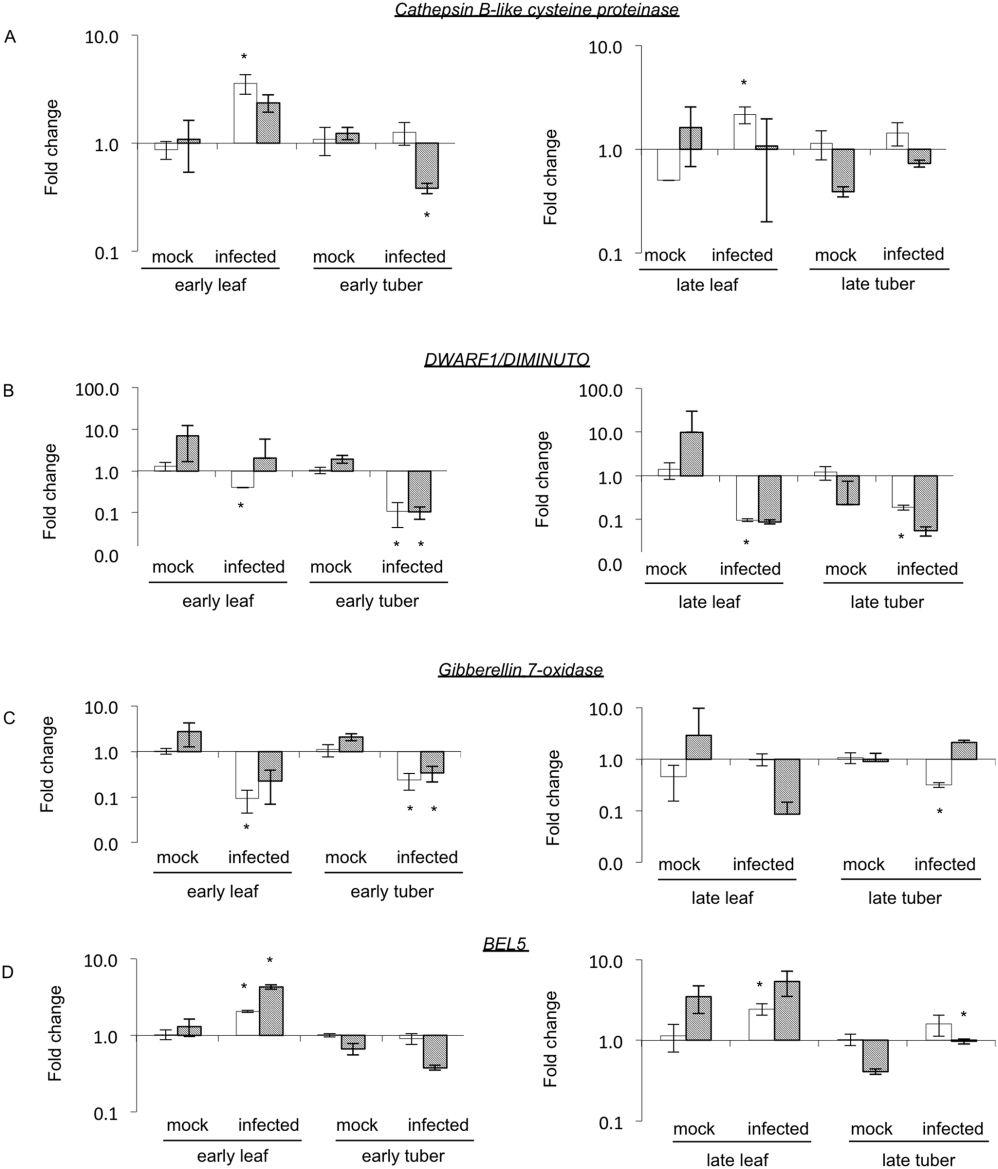
Quantitative RT-PCR analysis of candidate genes. Mock infected plants were used to normalize fold change of early and late samples in leaf and tuber tissues as indicated. White bars represent the RT-qPCR analysis; grey bars show the microarray data. Error bars represent standard error (SE). L23 and PP2A genes were used as internal normalization controls. Asterisk indicates that the means of fold change of the mock and infected samples are significantly different (Student’s t-test).

The candidate genes on chromosome 2 have diverse annotated functions. To validate the potato microarray data we have performed quantitative RT-PCR (RT-qPCR) to study the expression levels of the candidate genes in leaf and tuber tissues (Fig 4). Interestingly, on chromosome 2 under the tuber shape QTL, we found a gene with a role in defence responses: *Cathepsin B-like cysteine proteinase* [34]. This gene might contribute to the defence response upon viroid infection and not to tuber shape development. Its expression was increased in infected early leaf samples; this finding is in line with the microarray data but not in early tuber samples, although the microarray data suggested lower expression levels for this gene in infected tissues. In late leaf tissues the same expression was observed for *CBCP*, higher expression level in infected leaves compared to mock tissues and we could not observe change in the expression in late tuber samples (Fig 4A). Possibly the most interesting candidate is the *DWARF1/DIMINUTO* gene which has a role in plant sterol metabolism and can influence brassinolide levels, a hormone belonging to brassinosteroids (BRs). BRs have basic effects on growth and development of plants influencing the activity of many genes [35]. Our microarray data suggest a strong reduction in the expression of the *DWARF1/DIMINUTO* gene in early tuber samples upon viroid infection (S1 Table). RT-qPCR data supports this result showing about 8 times reduction of gene expression levels in early infected tissues (both leaf and tuber) and lower expression levels were detected in infected, late leaf and tuber samples compared to mock inoculated plants (Fig 4B).

Additionally, we have found in the dataset other genes which could influence tuber formation in potato. These genes show differential expression upon PSTVd infection but locate on other chromosomes, not under the chromosome 2 and 10 QTL. Although *Gibberellin 7-oxidase* was not reported to control tuberization in potato, other gibberellin oxidases have important roles in this biological process [22]. We found decreased expression levels of *Gibberellin 7-oxidase* in early leaf and tuber samples in infected tissues which is in line with the microarray data (Fig 4C). Although, there was no significant change for the late samples according to the microarray, we could detect decreased expression level in infected late tuber tissues. In potato, BEL5 is a transcription factor which interacts with the POTH1 protein promoting tuber formation [18]. *BEL5* RNA was described as moving into the stolon tip from the leaves to induce tuber formation. Our microarray data shows that in late tuber tissues there is an increased expression of *BEL5* in infected samples (S1 Table). In order to validate this data we performed RT-qPCR to investigate the expression levels in leaf and tuber tissues at the early and late stages of infection. Fig 4D shows significantly increased BEL5 RNA levels in both early and late infected, leaf tissues compared to the mock inoculated samples. We could not detect significant change in *BEL5* expression in early or late tuber tissues. It would be of interest to further investigate the translation of the above mentioned gene products in potato. However, the non-availability of specific antibodies currently prevented us performing such analysis (e.g. Western blotting).

Taken together, we validated the differential expression of some candidate genes suggesting their involvement in tuber formation.

## Discussion

Much is known about the structures, pathogenesis and symptoms of viroids in model plants and also some crops [36]. In our study we examined the effect of PSTVd infection on its natural host potato, since this host-pathogen interaction has not been characterised in detail [10]. Our aim is to gain more data about the pathogenesis in potato and to better understand the induced tuber phenotype; how tuber shape is changed and tuber formation is affected by PSTVd infection. Tobacco and tomato plants are usually more favoured to study this viroid primarily because of the shorter infection period and available genetic tools. A severe strain of PSTVd (KF440-2) was used to infect potato plants and study the effect on plant development and tuberization. Plants successfully infected by PSTVd showed typical symptoms (Fig 1A and 1B), altered foliage development including decrease in shoot and internode length, shortening of petioles and distortion of leaves similar to the symptoms observed in infected tomato [11]. The cultivar Safari was chosen for our experiments because we wanted to study the effect of viroid infection on formation of tubers and their shape. As Safari has round tubers it is easy to observe the spindle-tuber phenotype, as tubers become elongated following infection (Fig 1B). Typical infected tubers became elongated, sometimes lumpy and often with deeper eyes. We could observe a slight change in the skin colour: infected tubers had a lighter brown colour compared to the mock inoculated plants, although this phenotype could not be observed on every infected tuber. In contrast to other studies, we did not observe growth cracks on the tubers [10].

### A large-scale study of differentially expressed genes

Our study is a valuable resource for future studies aiming to find and analyse differentially expressed genes in potato induced by PSTVd infection, connecting pathogenesis and plant development. Previously, attempts were made for high-throughput analysis of gene expression using medium output macroarrays to identify differentially expressed genes at various stages of PSTVd infection in tomato [11]. Later, more advanced microarray analyses were conducted to gain better knowledge about differentially expressed genes in tomato upon viroid infection [12, 13]. Alternatively, high-throughput RNA sequencing techniques have been used to study expression levels of genes in different hosts. A particular example for this is high scale sequencing of small RNAs in order to identify, not only viroid derived small RNAs, but also endogenous small RNAs, including micro RNAs (miRNAs) [37]. It is worth noting that all these studies were carried out in tomato although there is an increasing demand on high-throughput experiments in potato. Previously, genome-wide expression studies were difficult to perform in potato. With the availability of the potato genome [38] a newly developed, custom 60k potato microarray was designed and used in our study which enabled us to analyse the expression pattern of virtually all predicted potato genes. Utilizing the low cost (compared to RNA sequencing) and robust analysis of the microarray, a genome-wide gene expression study was carried out at different stages of PSTVd infection in potato.

The 60k potato microarray is suitable to study different cultivars [39]. Genes show structured change upon PSTVd infection in leaf and tuber tissues (Fig 2). In our study we find very few genes which showed differential expression in more than one sample (early or late, leaf or tuber tissues) (S1 Table). Twenty genes show statistically significant differential expression in two samples (some of them belong to pathogenesis or hormone pathways) and there was no gene with significantly changed expression in three or four samples. The majority of genes seem to have altered expression usually only in one tissue and time point which might reflect the complexity of gene regulation upon development. The majority of differentially expressed genes in early leaf samples are up-regulated in infected tissues, whereas genes in late leaf tissues show mainly down-regulation (Fig 2A). In contrast, early tuber tissues often show down-regulation of gene expression at early time points and up-regulation of a larger number of genes at later time points of the infection. We speculate that this change might follow the movement of the viroid [10]. Viruses move from source to sink in the phloem, thus moving to the top of the plant invading newly emerging leaves and to the root, and in the case of potato into stolons and tubers [40]. Not surprisingly, we found among the differentially expressed genes defence or hormone pathway connected genes. Additionally, transcripts encoding heat-shock proteins were strongly up-regulated, and ribosomal proteins showed higher expression upon PSTVd infection [11]. Many visible symptoms, especially stunting and epinasty associated with viroid infection are indicative for altered hormone synthesis and metabolism [41]. Published microarray experiments in tomato using tolerant and sensitive cultivars showed many genes which had differential expression levels upon PSTVd infection [13], including genes encoding proteins associated with ribosomes, cell wall and plasma membrane. We found similar results in the case of potato infection: plant cell wall genes and genes connected to protein folding were significantly enriched in leaf samples (Fig 3A and 3B). Additionally, genes responding to stimulus, especially for oxidative stress were over-represented compared to mock infected plants for which response is similar to what was found in tomato [13]. In addition, different hormone signalling pathways (including GAs, BRs and Auxin) are influenced in tomato upon viroid infection; especially the Brassinosteroid-mediated signalling in viroid disease induction was shown [13], which is similar to our study. These data suggest that the visible symptoms of viroid infection could be strongly influenced by the affected hormone pathways.

### Tuber formation is affected by PSTVd infection

Although tuberization is one of the most important developmental processes in potato, it is not well understood. Tuber formation is regulated by different environmental conditions and hormones in plants. Species and cultivars from South America show strong regulation of tuberization by photoperiod. Short days induce tuber formation and some members of this pathway are known and characterized [14, 16, 42, 43]. However, in our study a long day adapted potato cultivar, Safari, was used which does not appear to be under the control of the photoperiod induced tuberization pathway. In addition to the day length control, different hormones including GAs, auxins and strigolactones were reported as having a role in tuber initiation and formation [21, 22, 24]. One of the main aims of our study was to identify differentially expressed genes in potato which can influence tuber formation upon viroid infection. Previously, we had examined tuber shape formation through a genetics study to identify QTL for length/width ratio of tubers [32]. In this current study we found several genes under the QTL connected to tuber shape which show differential expression upon viroid infection. On chromosome 2, we identified a pathogenesis related gene (*Cathepsin B-like cysteine proteinase*, *CBCP*), for which its expression increased in early leaf samples (Fig 4A). This gene is a good example of how regulation of defence related genes is changed upon viroid infection. As discussed above, genes connected to hormone pathways have significant enrichment in infected tissues. Interestingly, we found a gene (*DWARF1/DIMINUTO*) under the chromosome 2 QTL having a role in brassinosteroid signalling. This gene was described in Arabidopsis affecting plant development and fertility, *dim* mutants (mutation in *DIM/DWF1* gene) could be rescued by the addition of exogenous brassinolide [44]. In rice, mutation in a brassinosteroid-related gene can strongly influence plant development, showing a range of abnormalities in organ development and growth [45]. In early tuber tissues the *DWARF1/DIMINUTO* gene shows strongly reduced expression levels in infected tissues indicating a possible role of BRs in tuber shape development (Fig 4B). *DWARF1/DIMINUTO* could be the best candidate gene for further analysis to investigate its role in tuber shape formation or tuberization. Additionally, we found other genes which might have a role in tuber formation. *Gibberellin 7-oxidase* was shown as having decreased expression levels in infected early tuber tissues (Fig 4C). Although this gene was not reported before as having a role in tuberization, its reduced expression might indicate how GAs could be affected at different points of the pathway resulting in altered tuber development. Our study further supports the role of *BEL1-like* transcription factor (*BEL5*) in tuber initiation [18]. The mRNA of BEL5 gene can move in the plants from the leaves into the stolon tips, accumulate there and influence tuberization through binding to the promoter region of *ga20 oxidase1* (*ga20ox1*) [18, 46]. We found up-regulation of *BEL5* both in early and late leaf tissues showing the influence of this gene on tuber development (Fig 4D).

Taken together, our study shows that many genes have differential expression upon PSTVd infection in potato and PSTVd can be used as a tool to identify genes affecting tuber formation. Our experiment indicated commonalities but also some differences of viroid infection on plant development compared to other species (tobacco, tomato) showing the importance of defence and stress connected genes. Additionally, it brings light on the role of different hormone signalling pathways influencing symptom development. Our study further supports the role of known genes in tuber formation and points out the importance of GA and BR pathways in tuber/tuber shape development. A recent study suggests that BRs are master regulators of GA biosynthesis in Arabidopsis [47]. Our results, finding BR pathway genes (e.g. *DWARF1/DIMINUTO*) showing differential expression in viroid infected plants, supports the idea that the reason of altered plant development and tuber formation might be the mis-regulation of BR pathway genes and a direct or indirect effect on GA signalling. Further studies are necessary to clarify the role of the investigated genes in tuberization.

## Materials and Methods

### Plant material

*Solanum tuberosum L* ‘Safari’ was used for the experiments. Plants were grown from tubers in a glasshouse under long day (LD) conditions. Leaf samples (2^nd^-4^th^ leaves from the top) were taken to confirm PSTVd accumulation 9 wpi. ‘Early leaf’ samples were taken for gene expression studies at 14 wpi from leaves similarly to the 9 wpi sampling, and small tubers were harvested at the same time (‘early tuber’). ‘Late leaf’ samples were harvested from the same plants as described above at 21 wpi and whole tubers were collected at the same time for ‘late tuber’ samples.

### Viroid strain and infection

For potato infection the severe isolate of PSTVd^KF 440–2^ (GenBank accession number: X58388.1) was used. It was transcribed from an *EcoRI*-linearized pHa106 plasmid with SP6 RNA polymerase (NEB, USA)[33]. After the verification of the presence of viroid RNA by agarose gel electrophoresis, 150 ng transcript was used in mechanical infection (carborundum, Prolabo, VWR, UK) early in the morning. Infection of potato plants was carried out in insect-proof, air conditioned glasshouse in Heraklion, Crete, Greece.

### RNA analysis and gel blot assay

Leaf and tuber samples were homogenised under liquid nitrogen and total RNA was extracted from both tissues in RNA extraction buffer (38% saturated phenol, pH 5.2; 0.8 M guanidine thiocyanate; 0.4 M ammonium thiocyanate; 0.1 M sodium acetate; 5% glycerol v/v). Samples were cleaned with chloroform (Millipore, USA) followed by isopropanol precipitation. An extra phenol/chloroform step was introduced after the isopropanol precipitation in order to increase the purity of samples. Three μg total RNA was separated in a denaturing agarose gel (1.4% agarose, Invitrogen, USA; 0.7% formaldehyde, Millipore, USA) in order to test the integrity of RNA. Total RNA was transferred onto 0.45 μm nylon membrane (Whatman, GE healthcare, UK) for the detection of PSTVd transcripts. RNA (-) DIG labelled probe (DIG RNA labelling mix, Roche Diagnostics, Switzerland) was produced by T7 transcription from *HindIII*-cut pHa106 plasmid [33] and hybridization was performed overnight at 65°C in hybridization buffer (5x SSC; 1% SDS; 1x Denhardt’s; 250 mg/ml tRNA; 50% formamide). After three consecutive washes with 2x SSC/0.2% SDS at 65°C, CDP-star (Roche Diagnostics, Switzerland) was used for the detection according to the manufacturer instructions. Band intensity was quantified with the software Quantity One 4.4.1 (Biorad, USA) and values were normalised to leaf samples for each plant using ribosomal RNA (25S rRNA) as a control.

### Quantitative RT-PCR

One μg of each total RNA was used to prepare cDNA in a reverse transcription reaction (MMLV, Promega, USA) according to the manufacturer. Three technical replicates of each three biological replicates (for infected tuber tissue only two biological replicates) were used in subsequent Real-Time Quantitative Reverse Transcription PCR (RT-qPCR) reactions using FastStart Universal Probe Master with Rox (Roche Diagnostics, Switzerland). The Universal Probe Library system was used to design primers; its description and the details of RT-qPCR are described in Campbell et al., 2014 [48]. A StepOnePlus Real-Time PCR System (Applied Biosystems, Thermo Fisher Scientific, USA) was used for amplification with the following conditions: 10 min denaturation at 95°C followed by 40 cycles of 15 sec at 95°C, 60 sec at 59°C. Data was analysed as described in Hornyik et al., 2010 [49]. Since infection can influence internal reference controls, different independent reference genes were used and analysed (NormFinder, Best-keeper). L23 and PP2A were selected as having a p-value <0.05, thus being suitable for reference genes [50]. The average values of these genes were used in the quantification to normalise gene expression [51]. The PCR amplicons of the studied genes were confirmed by sequencing. The details of genes and primers are in S5 Table.

### Microarray analysis of gene expression and GO enrichment analysis

A custom Agilent microarray (Agilent Technologies, USA) was designed to the predicted transcripts from assembly v.3.4 of the DM potato genome as described [52]. A single-channel replicate block microarray design was utilised, the experimental design and complete datasets are available at ArrayExpress (http://www.ebi.ac.uk/arrayexpress/;accession E-MTAB-3869). RNA labelling and downstream microarray processing were performed as recommended in the One-Color Microarray-Based Gene Expression Analysis protocol (v.6.5, Agilent Technologies, USA) using the Low Input Quick Amp Labelling kit (Agilent Technologies, USA). Following microarray scanning using an Agilent G2505B scanner, data were extracted from images using Feature Extraction (v.10.7.3.1) software and aligned with the appropriate array grid template file (033033 D F 20110315). Intensity data and QC metrics were extracted using the FE protocol (GE1 107 Sep09). Entire FE datasets for each array were loaded into GeneSpring (v.7.3, Agilent Technologies, USA) software and data were normalised using default one-colour Agilent settings. Spot flags from FE (present or marginal) were used to remove probes with no consistent expression, leaving 25,291 probes. Statistical filtering was performed using volcano analysis (P-value ≤0.01, fold-change ≥ 2×). Data were visualised using gplot package (version 2.11.3) and a heatmap was generated using complete linkage as the agglomeration method and Pearson correlations to construct the distance matrices.

Gene Ontology (GO) enrichment of the gene lists was carried out using “TopGO” package (version 2.12) in R (v 3.1.0). The PGSC annotation (SolTub_3.0), as a dataset in ‘Plant Genes 45', was obtained from Gramene Plant Martview (http://ensembl.gramene.org/biomart/martview/), which provides the GO terms for each transcript ID. Probes were annotated with GO terms from the associated potato transcript IDs. In total, 142,686 probe and GO term relationships have been identified for 31,988 probes, which could be summarized into 3,766 GO terms. All probes on the microarray with GO annotations were used as background and genes with GO annotations from generated lists were investigated for enriched GO terms. Only GO terms with at least 5 annotated genes were considered. Fisher Statistics and weighted algorithm was used to calculate the p-values of significantly enriched GO terms. A p-value of 0.05 was used as cut-off to select GO terms.

## Supporting Information

S1 Fig PSTVd accumulation in infected plants. Viroid infection was confirmed by Northern blot analysis at 9 weeks post-inoculation (wpi). Methylene blue staining of ribosomal RNAs was used as loading control. WT1 sample is a non-infected control. Numbers represent individual infected plants.
(TIF)

S1 Table Microarray data of differentially expressed genes. Early and late samples of leaf and tuber tissues (as indicated on the separate sheets) are shown with raw and normalised expression levels and their ratio to mock infected plants. The samples compared are highlighted yellow. Additional information is included into the tables such as gene number, gene annotation, location, etc. The last sheet contains all the differentially expressed genes indicating in which samples they were found.
(XLSX)

S2 Table Complete set of microarray data for all the genes in potato. Normalised gene expression levels with standard error in early and late leaf and tuber tissues. Systematic column contains the unique identifier of probes; primary accession is the gene transcript number of potato genes. Functional annotation is included with gene location data (superscaffold, chromosome and gene IDs: peptide, gene, CDS and transcript). GO categories are included (MpMan bin, MapMan name) with description.
(XLSX)

S3 Table GO term enrichment of different categories. Biological process, Cellular component and Molecular function GO term categories are shown on separate sheets. Values in the tables represent the p-values of enrichment in different samples as indicated.
(XLSX)

S4 Table Gene list of three superscaffolds under the tuber shape QTLs. Gene list of three superscaffolds where tuber shape QTL are mapped. Gene location, gene number and annotated function are indicated. Genes highlighted by green are differentially expressed upon PSTVd infection according to the microarray hybridization. The highlighted candidate genes were extracted into Table 1.
(XLSX)

S5 Table Primers used in this study. List of primers which were used in this study with the corresponding transcript number, sequence and UPL probe number.
(XLSX)
