# Supplementary figures and images for: Insight on Genes Affecting Tuber Development in Potato upon Potato spindle tuber viroid (PSTVd) Infection
Source: PLoS One. 2016 Mar 3;11(3):e0150711. doi: 10.1371/journal.pone.0150711 (PMC4777548; doi:10.1371/journal.pone.0150711)

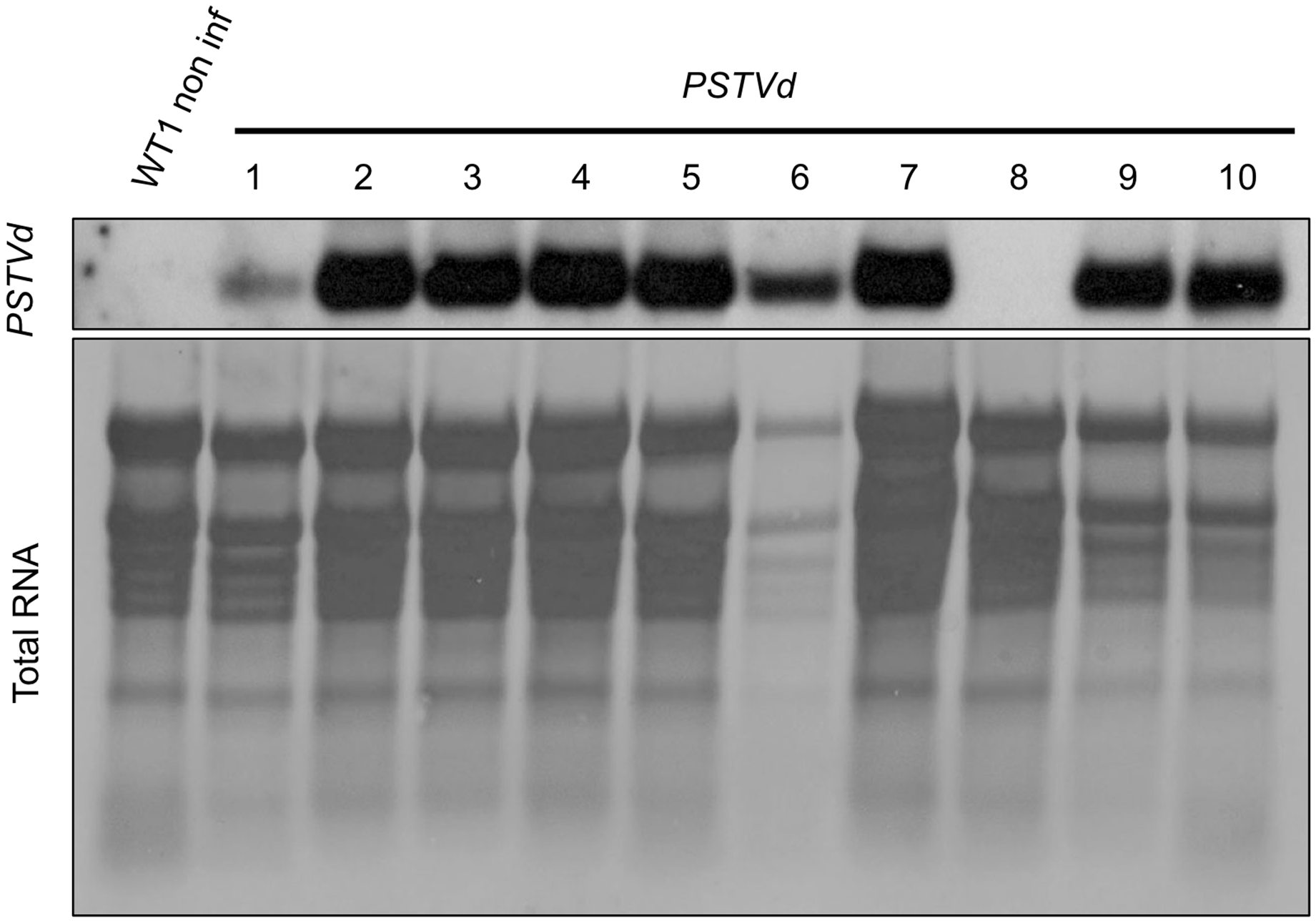

Supplement: S1 Fig — Viroid infection was confirmed by Northern blot analysis at 9 weeks post-inoculation (wpi). Methylene blue staining of ribosomal RNAs was used as loading control. WT1 sample is a non-infected control. Numbers represent individual infected plants. (TIF) [file pone.0150711.s001.tif]
